# A Delay in the Identification and Diagnosis of Polycythaemia Vera in a Patient Presenting With Chest Pain: A Case Report

**DOI:** 10.7759/cureus.110119

**Published:** 2026-06-02

**Authors:** Divneet Kaur Chadha, Mehul Panicker, Medha Panicker

**Affiliations:** 1 Medicine, Glan Clwyd Hospital, Bodelwyddan, GBR; 2 Medicine, Furness General Hospital, Barrow-in-Furness, GBR

**Keywords:** chest pain, cytoreductive therapy, leukocytosis, polycythaemia, polycythaemia vera, splenomegaly, thrombocytosis

## Abstract

Polycythaemia vera (PV) is a chronic myeloproliferative neoplasm characterised by proliferation of erythroid lineages leading to erythrocytosis and can also present with variable proliferation of myeloid and megakaryocytic lineages causing leukocytosis and thrombocytosis. PV is strongly associated with mutations in the JAK2 gene with the mutation being found in >95% of patients with PV. It can present with splenomegaly, which tends to cause abdominal pain. Diagnosis is based on the WHO criteria, which is based on haemoglobin and haematocrit levels, the presence of JAK2 mutations and erythropoietin levels with bone marrow changes. Typically, PV is managed with low-dose aspirin to prevent thrombotic complications, venesection to control haematocrit and cytoreductive therapy. This case discusses an unusual presentation of a patient with polycythaemia vera who presented with chest pain, leading to delays in diagnosis.

## Introduction

Polycythaemia vera (PV) is a myeloproliferative neoplasm characterised by clonal overproduction of erythrocytes, which can be accompanied by white cell and platelet proliferation [1]. The median age of presentation is around 60 years and men are slightly more affected than women [2]. PV can present with splenomegaly due to extra-medullary haematopoiesis, which can cause abdominal pain [1]. Another risk of PV is the increased risk of thrombosis. One in five patients with PV presents with a thrombotic event as their first presentation, which significantly contributes to morbidity and mortality. Early diagnosis and management can help minimise the long-term complications from arterial and thrombotic events and improve outcomes [2].

In this case, the patient had splenomegaly, which caused chest pain and abdominal discomfort, leading to the working diagnosis of chest infection for which the patient repeatedly received antibiotics to no avail. The patient underwent further testing, which was positive for JAK2 mutation, and went on to receive venesection and subsequent hydroxycarbamide therapy. This report highlights the importance of a thorough physical examination and varied differential diagnoses to prevent delays in patient care.

## Case presentation

History of presenting complaint 

A woman in her fifties presented to the emergency department (ED) for the third time in a month with pleuritic lower left chest pain. She had initially presented to her general practitioner (GP) six weeks ago and was investigated over the past six weeks in GP and in the ED. She had no previous history of thrombosis and was not a smoker. She had undergone multiple chest x-rays as well as a CT pulmonary angiogram, which was negative for a pulmonary embolism but showed minor atelectasis/consolidation at the left lower lung base. She received three courses of antibiotics over six weeks, including amoxicillin, doxycycline and co-trimoxazole for the consolidation seen on imaging.

Case presentation and progression 

Two days after starting co-trimoxazole, the patient attended ED with widespread erythematous raised lesions all over her torso, arms, and legs. She noted that she had been experiencing pruritus when taking hot baths for the past few weeks.

**Figure 1:**
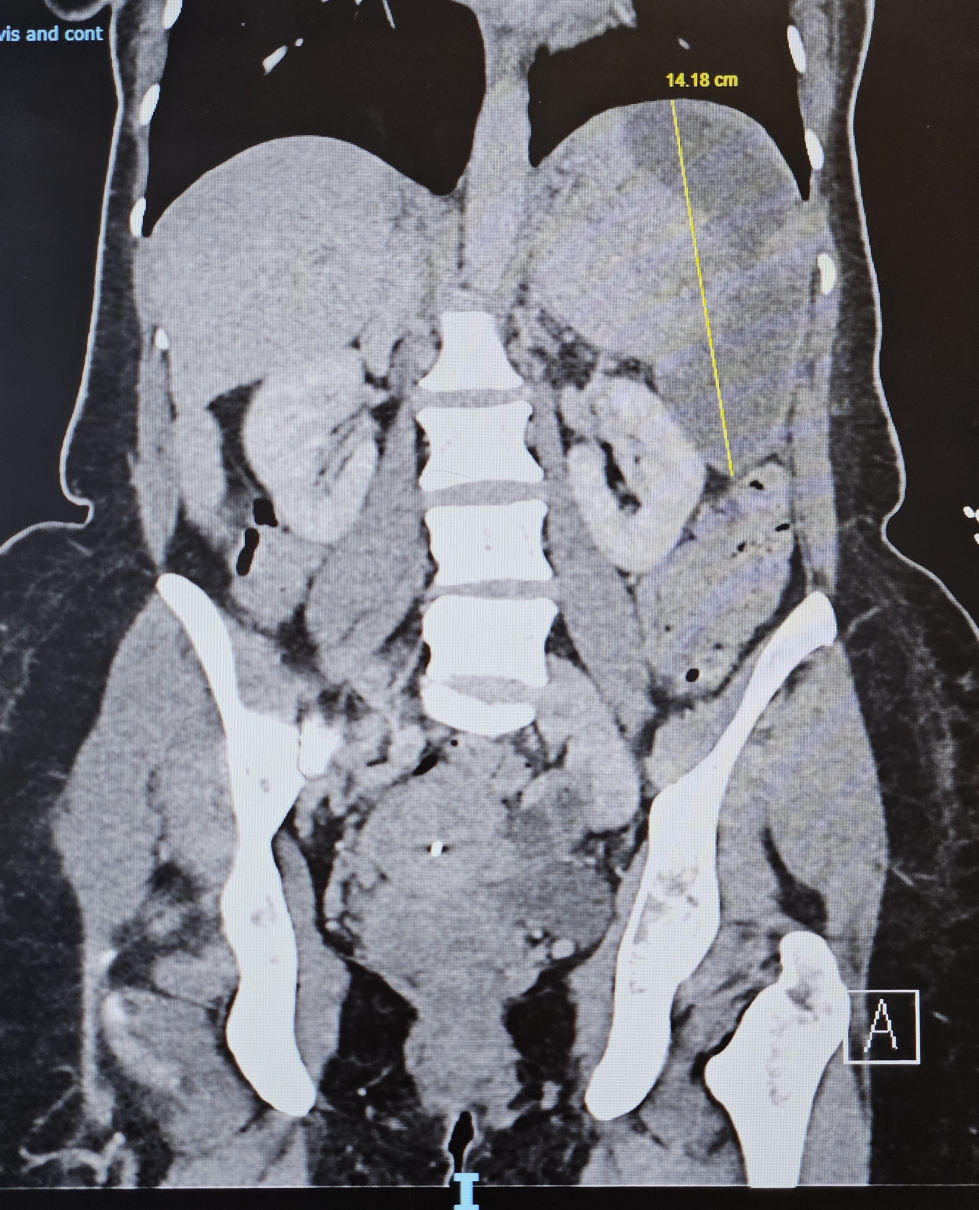
A 14 cm splenomegaly visible on CT abdomen and pelvis

On examination, the patient had massive splenomegaly, which was confirmed with ultrasound and CT scans (Figure 1). The CT scan showed the spleen measured 14 cm with multiple splenic infarcts without hepatomegaly. Blood tests on this admission showed a haemoglobin of 175 g/L and white cell count of 40.1x10^9^/L, with platelets of 813x10^9^/L. As seen in Table 1, her white cell count had dramatically jumped in 24 hours since her previous visit to the GP. She received intravenous antibiotics to cover for Stevens-Johnson syndrome (SJS).

**Table 1:** Trend of full blood count over multiple presentations to GP and ED GP: General physician; ED: emergency department.

Date and Location	Haemoglobin (g/L)	Haematocrit (L/L)	White Cell Count (x10^9^/L)	Platelets (x10^9^/L)
4 February at GP	178	0.59	12.6	550
6 February at ED	180	0.60	14.9	578
18 February at ED	188	0.61	18.5	651
27 February at GP	197	0.66	27.8	904
28 February at ED	183	0.55	35.9	865

In retrospect, it was noted that the patient had polycythaemia, leukocytosis and thrombocytosis since she was first investigated for chest pain at GP, as seen in Table 1. She had also been persistently hypertensive during the admission despite being on amlodipine and doxazosin. JAK2 and erythropoietin levels were in progress as requested by a medical consultant who noted the persistent panmyelosis on a previous admission. A referral to haematology was made regarding the initiation of aspirin to prevent clots and to discuss further management. Dermatology and ophthalmology reviewed the patient for possible SJS secondary to co-trimoxazole, which improved with topical treatments and eye drops. She was discharged after a week with outpatient follow-up with haematology for polycythaemia vera.

Outcome and management

Upon discharge, the patient’s JAK2 analysis from blood samples returned positive with a mutation at c.1849G>T, resulting in the protein change V617F at 41% variable allele frequency. Her highest recorded haematocrit was 0.66 L/L, and her erythropoietin level was <5 mU/mL. A bone marrow biopsy was performed, which detected actionable variants in the JAK2 (46% allele frequency), EZH2 (2% allele frequency) and TP53 genes (2% allele frequency). No further workup was done for thrombophilia or other hypercoagulable states. She was started on long-term aspiration and received four sessions of venesection, which brought down her haematocrit to 0.46L/L. As she had presented with splenic infarcts, she was considered high risk for thrombosis [3]. Combined with the leukocytosis, thrombocytosis and raised haematocrit, cytoreductive options were discussed with the patient who declined Pegylated (PEG) interferon and instead opted for hydroxycarbamide.

## Discussion

Polycythaemia vera (PV) is a progressive myeloid malignancy that falls under a group of disorders called myeloproliferative neoplasms. It is characterised by a gain of function mutation in the JAK2 gene, which is present in >95% of patients with PV [4]. This mutation leads to the overactivation of the JAK-STAT pathway that overstimulates erythroblastic lineage [5]. It typically results in panmyelosis as it concomitantly affects both megakaryocytic and myeloid cell lineages [1,6].

The advancement of PV is categorised into three distinct phases: the proliferative phase, the stable phase, and lastly the “spent” phase [6]. The proliferative phase is the initial onset of symptoms. Symptoms stem from a hypercoagulable state and are non-specific in nature. Neurological symptoms include vertigo, tinnitus, headache and altered vision. Aquagenic pruritus and erythromelalgia are uncommon but sensitive markers. Haemorrhagic and thrombotic complications are seen in a minority of patients and affect various organ systems to cause venous thrombotic events, Budd-Chiari syndrome and ischaemic cerebrovascular accidents. Gastrointestinal complaints are more frequently encountered and present with abdominal pain or early satiety, commonly indicative of peptic ulcers or splenomegaly [7].

Diagnosis 

As previously mentioned, the patient had panmyelosis for six weeks with raised haematocrit, which was treated as reactive with courses of antibiotics, but no further investigations were done to exclude malignancy. 

Current guidelines established by the World Health Organization in 2016 categorise diagnostic requirements for PV into major and minor criteria [8]. 

Major Criteria

(1a) Haemoglobin (Hb)>16.5 g/dL in men or >16 d/dL in women OR; (1b) Haematocrit (HCT) > 49% in men or >48% in women OR; (1c) Increased red cell mass by 25% above mean normal predicted value. 2. Bone marrow biopsy showing hypercellularity for age with panmyelosis including prominent erythroid, granulocytic and megakaryocytes proliferation with pleomorphic, mature megakaryocytes (variation in size). 3. Evident mutation in JAK2V617F or JAK2 exon 12.

Minor Criteria

1. Decreased serum erythropoietin levels: Fulfilment of all three major criteria or a combination of the first two and the minor criterion allows confirmation of PV. In exceptional cases, bone marrow biopsy may be omitted if major criterion 3 and the minor criterion met in the presence of marked erythrocytosis - defined as Hb>18.5 g/dL or HCT>55.5% in men and Hb>18 g/dL or HCT>55% in women [8].

The patient fulfilled the first two major criteria (1 and 3) and the minor criteria on this admission.

Management

Prophylactic treatments for thrombotic complications are guided by risk-based stratification using patient demographics. Cardiovascular risk factors for thrombosis should be controlled using lifestyle modifications regardless of the risk category. Patients are at low risk for thrombosis if <60 years old with no history of thrombosis. Venesection is used therapeutically to remove excess erythrocytes. Aspirin may be given up to two times a day with doses ranging from 75 to 100 mg. The patient was started on 75 mg aspirin twice daily as per guidance. In high-risk patients (age >60 years with a history of thrombosis), augmentative cytoreductive therapy is administered with hydroxyurea. The second-line therapies include pegylated interferon or busulfan [1,9]. Surgical interventions such as splenectomy and shunt placement are indicated in the presence of splenic infarcts or altered hepatic vasculature [10]. Remission of symptoms marks the onset of the stable phase.

The “spent” phase is a progression of PV characterised by transformation into secondary conditions such as myelofibrosis, acute myeloid leukaemia, and myelodysplastic syndrome [1].

## Conclusions

This report presents a case of a patient who presented with chest pain with underlying PV and panmyelosis as well as splenomegaly with multiple splenic infarcts compounded by SJS. A thorough examination during the patient’s first presentation might have led to the splenomegaly being detected and being considered earlier while forming differential diagnoses, given her history of persistently raised haemoglobin. This may have led to an earlier CT scan of the abdomen and consequent referral to haematology, initiating further investigations into polycythaemia vera as well as its treatment.
